# The effects of simulated monocular and binocular vision impairment on football penalty kick performance

**DOI:** 10.1002/ejsc.12145

**Published:** 2024-06-08

**Authors:** Harrison K. Leivers, Peter M. Allen, Matthew A. Timmis, Franzi Zenk, Jaspreet Uppal, Oliver R. Runswick

**Affiliations:** ^1^ Cambridge Center for Sport and Exercise Sciences (CCES) Anglia Ruskin University Cambridge UK; ^2^ Vision and Hearing Sciences Research Center Anglia Ruskin University Cambridge UK; ^3^ Department of Psychology Institute of Psychiatry, Psychology and Neuroscience King's College London UK

**Keywords:** classification, football, minimum impairment criteria, para‐sport, soccer, visual impairment

## Abstract

Sports performance is relatively robust under high levels of binocular blur. However, the limited research studies investigating monocular impairments has shown it has a larger impact on sport performance. This research study is relevant for classification in sports for athletes with vision impairment (VI), where visual acuity (VA) from the better eye is used during classification. Across two experiments, we aimed to establish the point at which binocular and monocular impairments affected performance in a football penalty kick (PK) through simulating varying severities of degraded VA and contrast sensitivity (CS) in active football players. In experiment one, 25 footballers performed PKs as VA and CS were systematically decreased in both eyes, and in one condition, visual field (VF) was reduced. The most severe VA/CS condition and reduced VF significantly impacted outcome, ball velocity and placement (ball kicked closer to the centre of the goal) (*p* < 0.05). In experiment two, 29 different footballers performed PKs as VA and CS of only the dominant eye were systematically decreased and in one condition the dominant eye was occluded, and participants viewed their environment through the non‐dominant eye (monocular viewing). No differences were observed when assessing monocular impairments influence on outcome, velocity and ball placement. PKs have a high resilience to VI, but binocular impairment has a more immediate effect, suggesting binocular measures should be used in classification processes in football.

## Highlights

- Penalty kick (PK) performance was relatively robust to severe artificial binocular visual impairment. Performance reduced at 1.96 logMAR, causing the ball to be kicked slower (lower velocity) and closer to the centre of the goal.
- Monocular viewing or impairment did not influence the velocity at which the ball was kicked or where the ball was placed. Therefore, PK performance was maintained at the habitual level.
- The evidence suggests that PK performance is more sensitive to binocular impairments than monocular impairments. Therefore, it may be beneficial for footballers with vision impairment to be classified using binocular assessments.

## 1 INTRODUCTION

A wide body of work has investigated the role of visual function in sports performance. In football, the visual system enables athletes to distinguish between team members and opponents, identify stimuli outside the central vision and estimate relative depth, influencing the judgement of player and target location (Millard et al., 2022). Understanding the relationship between vision and performance is critical for classifying athletes with a vision impairment (VI), where the outcome of competition should be determined by an athlete's ability rather than their impairments. Therefore, classification aims to group individuals of a similar level of activity limitation (Mann & Ravensbergen, 2018), and research into the relationship between impairment and performance is required to support this process. Currently, VI football classification is conducted through monocular assessment, and competitors are classified based on their best‐performing eye. However, empirical evidence has suggested that monocular and binocular impairments may have different effects on performance (Bulson & Ludlam, 2009; Vera et al., 2020) and expert consensus in VI football (Runswick et al., 2021) and VI sport more broadly (Mann & Ravensbergen, 2018) has suggested binocular visual assessment would be more beneficial when classifying athletes.

Currently, VI football is comprised of two classes: ‘Blind Football’ (B1), which competes within the Paralympic games, and ‘Partially Sighted’ (B2/B3), which is currently ineligible for Paralympic competition. Each form of VI football has different eligibility criteria, and allocation depends on one's level of vision. B1 competitors are required to have a visual acuity (VA) >2.6 logMAR. Competitors are classified as B2 if their VA ranges from 1.5 to 2.6 logMAR and/or their visual field (VF) <5°, or as B3 if their VA ranges from 1.0 to 1.4 logMAR and/or VF 5–20°. Both forms of the game play adapted versions of futsal. B1 football is subject to several adaptations, such as a sounded ball, and shouts of ‘voy’ (enables an estimate of player location); whilst players receive verbal instruction from guides positioned in each third of the court, players accrue sensory information through auditory sources. Players wear blindfolds and kickboards are located on the side of the pitch. Currently, VI football classes are not based on sport‐specific evidence and are, therefore, non‐compliant with the classification code. Equitable competition should be achieved by developing sport‐specific classifications through empirical evidence understanding VIs influence on performance (IPC, 2015).

Simulation studies are one of the many methods that have been used in classification research, often implemented when determining a sports minimum impairment criteria (MIC) (Mann & Ravensbergen, 2018). The MIC represents the degree of impairment that impacts performance in the unadapted version of a sport; in the case of VI football, this is futsal (IPC, 2015). Simulation studies systemically induce VI in fully sighted individuals using filtered glasses, contact lenses or computer‐based simulations and aim to identify the point at which performance significantly deteriorates within a given sport. The use of simulations to impair visual functioning has consistently shown that athletes maintain performance across a variety of tasks until relatively severe impairments (Bulson et al., 2015; Mann et al., 2007, 2010; Runswick et al., 2023; Vera et al., 2020).

Previous research has artificially induced VI by reducing (VA; the spatial resolving capacity that refers to the sharpness/resolution of the central fixation); a higher logMAR value denotes inferior resolution. For example, basketball (Bulson et al., 2015; Vera et al., 2020), golf (Bulson et al., 2008), judo (Krabben, Mann, et al., 2021), shooting (Allen et al., 2016), football (Runswick et al., 2023) and cricket (Mann et al., 2007, 2010) have all shown that performance is resilient to relatively high levels of binocular impairment. In football, specifically, Runswick et al. (2023) used simulation glasses to systematically reduce VA and contrast sensitivity (CS, the ability to discern or distinguish between different luminance levels in static images). Skilled players completed the visually impaired football skills (VIFS) test, which measures dynamic technical football performance (movement around the court, ball control, dribbling and passing) (Runswick et al., 2023). Participants were able to maintain dynamic football performance at around 1.0–1.2 logMAR VA. However, this study only included binocular blur conditions and did not include visual field (VF the entire area that can be seen by the eye when fixating at a point) or assess the potentially important skills of shooting or set pieces (Runswick et al., 2021).

Self‐paced sporting motor skills (shooting, free throws, putting) can be initiated when the individual has achieved optimal mental readiness and subsequently could be less sensitive to VI (Allen et al., 2018; Bulson et al., 2008, 2015; Williams et al., 2004). This is due to blur adaptation, where individuals experience a relatively rapid recovery in visual resolution with a concurrent reduction in perceived blur (Bulson et al., 2008) and extract sufficient visual information. However, in self‐paced tasks such as basketball free throws (Bulson et al., 2015) and golf putting (Bulson et al., 2008), performance was maintained at 1.40 and 2.00 logMAR, respectively. The effects of degraded CS may also differ in self‐paced and dynamic tasks. CS is a strong indicator of how individuals with low vision manage in activities of daily living (driving and avoiding falls) and was deemed a strong indicator of shooting performance (Allen et al., 2018). However, initial evidence suggests that CS is a poor predictor of football performance (Runswick et al., 2023), and a similar relationship was identified within judo (Krabben, Ravensbergen, et al., 2021). These studies equally impaired each eye simultaneously.

Despite many studies examining the effects of binocular blur, there is little research in a sporting context assessing individuals who view their environment through two dissimilar monocular images (if the athlete is amblyopic resulting from strabismus, anisometropia or aniseikonia) (Vera et al., 2020). This is relevant for paralympic classification, where athletes have historically been classified through their best‐performing eye. This is particularly controversial within VI sports as current classification procedures may not adequately capture the amount of vision used in competition (Mann & Ravensbergen, 2018), as athletes typically use both eyes. In an initial expert consultation, VI football experts agreed that competitors should be classified through the assessment of both eyes together (Runswick et al., 2021).

Previous research studies have applied artificial monocular impairments by occluding (monocular viewing) and blurring the dominant eye (monocular impairment). Vera et al. (2020) reported significantly poorer free throw performance during monocular impairment and viewing compared to habitual performance and binocular impairment. Moreover, golf putting was significantly poorer under monocular viewing conditions irrespective of putt distance when putting at a 3 cm target (Bulson & Ludlam, 2009). These performance decrements have been attributed to reduced stereopsis (depth perception), which is the ability of the visual system to use horizontal retinal‐image disparity information to perceive relative depth and three‐dimensional shape (Bulson & Ludlam, 2009). Accurate object depth determination is a major advantage of binocular vision, as the ability to judge depth is attributed to the disparity of each retinal image; consequently, under monocular viewing absolute object depth cannot accurately be determined (McCoun & Reeves, 2010). Due to binocular disparity being unavailable, monocular cues are not sophisticated enough to accurately determine a target's absolute depth (motion parallax, linear perspective, relative size, shading, shadows and texture gradient) (Vera et al., 2020). To date, no research study has investigated self‐paced elements of football performance (such as penalty kicks) or the effects of monocular versus binocular impairment. To inform classification in VI football, research is required to understand these aspects of the performance‐impairment relationship.

The study aimed to identify if futsal penalty kick (PK) performance is affected by binocular and monocular VI and, if so, document the earliest point at which velocity, placement, and overall penalty performance deteriorates. Previous research has shown that the ball's speed (velocity) combined with the location where the ball is kicked within the goal impacts PK outcome (Timmis et al., 2014). This will add to the evidence base aiming to develop the MIC for VI football. For experiment 1, artificial binocular blur was systematically applied to participants, enabling varying degrees of simulated VI to be compared with habitual kick velocity, placement and performance. It was hypothesised that all variables would have high resilience to blur, but velocity would decrease, and placement would become more central to minimise potential errors, eventually leading to overall performance decrease (Bulson et al., 2008, 2015). For experiment 2, the same dependent variables and artificial impairments were simulated but only on the dominant eye. It was hypothesised that monocular viewing would negatively impact velocity, placement and performance earlier than artificial binocular impairment due to a reduced ability to perceive depth (Bulson & Ludlam, 2009; Vera et al., 2020).

## 2 EXPERIMENT 1 METHOD

### 2.1 Participants

Twenty five participants with a mean age of 22 ± 3 years were recruited to participate in the study. To be eligible for participation in the study participants were required to be active footballers that were in the university (futsal or football) teams or competing in organised leagues. Participants were deemed ineligible if they wore glasses or were injured. Participants were sports students who competed in the university's amateur futsal/football clubs. The university ethics panel ethically approved the study. Written informed consent was obtained and the research was conducted in accordance with the principles of the Declaration of Helsinki. Twenty‐two males and 3 females were recruited. The sample size was planned based on an a priori sample size calculation with the resources constraints inherent with recruiting footballers (Lakens, 2022) to detect a medium effect (*f* = 0.25) with two‐tailed *α* of 0.05, power (1 − *β*) of 0.80 across six repeated measures (simulation levels) with a moderate correlation between measures (0.5) requiring a minimum sample size of 24. This calculation was used for experiments 1 and 2. We considered the use of Vera et al. (2020) large effect size for this calculation, but task and study design were considered too far from the constraints on a PK for this to produce a similar effect. Therefore, we chose to recruit a feasible sample based on a medium effect. Female and male players were included as classification evidence must reflect the impairment–performance relationship of the men's and women's games. We did not hypothesise that gender will impact the impairment–performance relationship (Runswick et al., 2023), and as previously evidenced in VI Judo (Krabben, Mashkovskiy, et al., 2021). The women's game has recently been introduced at the 2023 IBSA world games, where 22 percent of footballers were female, indicating that our sample is an accurate representation of the study's target population.

### 2.2 Protocol

A block randomised study design where each block (simulation level) entailed three PKs; in total participants took 24 PKs and each block were completed before moving on to the next. To determine habitual performance (control condition), participants took x3 PKs under standard binocular viewing conditions (no manipulation of participant vision). The establishment of habitual performance is in line with previous research studies (Timmis et al., 2014).

To account for learning and order effects the order of simulation level was randomised between participants.

Before the PK, participants were instructed to kick the ball into an area they believed the goalkeeper could not reach when diving. This avoided the use of the keeper‐dependent strategy (Wood & Wilson, 2010) or pausing using the deceptive strategy during the run‐up (Wood & Wilson, 2010). Goalkeepers were positioned in the centre of the goal, with their arms outstretched and remained in the same position until the football had been kicked. These instructions were reinforced before each protocol initiation, this approach was successfully implemented, and no PKs were retaken. This ensured performance was not impacted through altered task demands between participants. Two goalkeepers of similar experience (actively competing in organised amateur leagues) were used during data collection. The same goalkeeper was used for every PK within a participant's dataset (goalkeeper only changed between participants). Goalkeepers only changed between participants when the primary goalkeeper was not available.

### 2.3 Apparatus

Penalties were taken indoors and following The Football Association (F.A.) guidelines for 5, 6 and 7‐a‐side indoor football (The F.A., 2012). A size 4 football (Mitre super league indoor football) was placed on a penalty spot 6 m from the centre of a goal measuring 3 m wide by 2 m high. To ensure the goalkeeper's safety when diving for the ball, gym mats (0.03 m thickness) were placed in front of the goal to prevent goalkeeper injury.

A Photon Fastcam (Ultima 512) high‐speed video camera was placed (at a height of 0.3 m) perpendicular to the penalty spot (distance of 1.5 m) to record (at 250 Hz) the displacement of the football once kicked. Data recorded from the high‐speed camera were analysed using WinAnalyze software (Mikromak) to calculate initial velocity of the football when kicked. The resultant (product of both horizontal and vertical) kicking velocity was calculated.

A Canon (Legria HD HF R28) video camera was positioned 10 m from the goal (behind the penalty taker) to record (at 25 Hz) the end location of the football after the penalty had been taken (either within the goal, irrespective of whether the penalty was saved or not, or wide of the goal on the wall behind the goal). Screenshots from the video identifying the football's end location were taken using XnView (Ver. 1.99.6) and uploaded into Didge (Image Digitizing Software Ver. 2.30b1) to allow the horizontal and vertical end location of the football to be defined.

### 2.4 Simulation of VI

Following pilot testing, safety glasses produced by Bollé Safety, UK, were chosen as mounts for simulation lenses due to their robustness to movement. Hampshire Frost filters produced by Lee Filters were selected as these are the same as those used in the established ‘Cambridge simulation glasses’ (Goodman‐Deane et al., 2013). We developed a systematic decrease in VA and CS through a combination of filters, these were identical to those used in Runswick et al. (2023). Table 1 shows the final seven levels of simulation.

**TABLE 1:** Level of simulated impairment used in this study.

Level	How simulated	Mean visual acuity logMAR (ETDRS)	Mean contrast sensitivity logCS (MARS)
Habitual	No simulation	−0.04 ± 0.17	1.78 ± 0.06
Level 1	Four layers of 258	0.52 ± 0.17	0.83 ± 0.11
Level 2	Five layers of 258	0.72 ± 0.16	0.65 ± 0.13
Level 3	Six layers of 258	1.03 ± 0.14	0.40 ± 0.15
Level 4	Seven layers of 258	1.30 ± 0.13	0.15 ± 0.11
Level 5	One layer of 256	1.61 ± 0.09	0.01 ± 0.03
Level 6	One layer of 253	1.96 ± 0.10	0.00 ± 0.01
Level 7	Visual field approx. 5–9° (Timmis et al., 2021), but VA and CS remain habitual.		

VA was assessed using an externally illuminated Tumbling E ETDRS chart at 4 m (Precision Vision, Chart 1) and recorded in logMAR. Letter‐by‐letter scoring was used; higher logMAR values indicate a poorer central vision function. In instances of extremely low vision, where vision was degraded to the point of the illiterate ETDRS chart being unidentifiable, the Berkeley Rudimentary Vision Test (BRVT; Bailey et al., 2012) was used.

CS was measured in logCS with a higher value indicating a superior function. The MARS number test (Mars Perceptrix) measured CS. This test consists of three charts, each with eight rows of six numbers, starting (top left) with the highest contrast of 1.92 logCS and a reduction of 0.04 logCS per number. Participants were instructed to read each number, and the test was terminated following two incorrect answers.

### 2.5 Data analysis

Football end location: The bottom centre (middle) of the goal was defined as the ‘0’ horizontal and ‘0’ vertical coordinate. Values exceeding 1.5 m in the horizontal or 2.0 m in the vertical direction were excluded from the analysis as ‘missed’ penalties. Absolute horizontal end ball location was used to remove negative coordinates from the analysis. Horizontal and vertical end locations were analysed separately.

### 2.6 Statistical analysis

A separate one‐way ANOVA was conducted to measure the success of the VA and CS manipulation. A Pearson (*r*) correlation coefficient assessed the VA and CS relationship.

#### 2.6.1 Repetition

Friedman's analysis was used to investigate whether performance altered due to protocol familiarity (repetition effect; x3 PKs per block). Each trial repeat was collapsed across vision conditions; the effect size was calculated using Kendall's coefficient of concordance (*W*).

#### 2.6.2 Order effect

An average of the three trial repeats for the first (starting block), fourth (middle block) and eighth (last block) testing condition was analysed using Friedman analysis, with planned follow‐up post hoc testing using a Bonferroni corrected Wilcoxon Signed Ranks test; the effect size was calculated using Kendall's coefficient of concordance (*W*). The first, fourth and eighth blocks were different (e.g. level of blur) between each participant due to a block‐randomised study design being implemented. All follow‐up post hoc tests were compared to the Habitual vision condition and there was no other between‐vision condition comparison.

#### 2.6.3 Performance, initial ball velocity and ball placement

The number of penalties scored and not scored (saved or missed combined), initial ball velocity and endpoint location (X: side‐to‐side and Y: vertical) across Vision condition (x8 levels) were analysed using repeated measures ANOVA as within‐subject factors; follow‐up post hoc testing was completed with a Bonferroni corrected *p*‐value to account for multiple comparisons. Effect sizes were calculated using Partial Eta squared ($\eta_{p}^{2}$), and post hoc effect sizes were calculated using Cohen's *d* (*d*).

#### 2.6.4 Receiver opertator characteristic curves

Receiver operator characteristic (ROC) curves were built to determine which VI severity affects performance. ROC analysis was conducted in Microsoft Excel, where VA and CS were examined. ROC curves are developed by plotting the true positive rate (sensitivity) and the true negative rate (specificity). The true positive rate is defined as the proportion of positives which are identified correctly, referred to as the probability of a positive test, whereas the true negative rate is the proportion of negatives which are identified correctly, referred to as the probability of a negative test (Carter et al., 2016). Identifying a meaningful difference determines whether a data point is classified as a true positive or a true negative. To establish an optimal cut‐off, a meaningful difference was defined as the first statistically significant performance reduction when comparing each simulation level to habitual performance—in this instance, 18% (occurred at level 6). This enabled performance to be categorised into ‘expected’ and ‘below expected’. A good sensitivity cut‐off will include players who perform below expected (indicating Impairment affects performance), and a good specificity would exclude players who are not performing worse than expected (Indicating that Impairment does influence performance) (Runswick et al., 2023). The Youden's *J* (Equation: Youden's *J* = sensitivity + specificity − 1) statistic measured the balance between sensitivity and specificity.

#### 2.6.5 Decision tree analysis

Decision tree analysis was used to identify an optimal MIC, accounting for VA and CS, the tree‐based classification model was applied (‘tree’ package in R) (Zhang, 2016). The method implemented recursive binary splitting, allowing the best split in performance to be continuously made. An optimal separation of ‘expected’ and ‘below expected’ performance would result in two nodes. Following the initial split, this process continued until the stopping criterion was attained, requiring a minimum of five data points per node. The decision tree was then pruned through cross‐validation, determining the optimal tree complexity. The tree was built and pruned using the data from each simulation as the training data set. ROC curves and decision tree analysis were conducted if VI significantly impacted performance. This evidenced through the ANOVA analysis.

## 3 EXPERIMENT 1 RESULTS

### 3.1 Simulated impairments

Simulation level significantly affected VA (*F*(6, 114) = 680.892, *p* < 0.001, $\eta_{p}^{2}$ = 0.973). Post hoc comparisons showed a significant difference (all *p* < 0.01) between all levels of simulation. There was a significant effect of simulation level on CS (*F*(6, 114) = 1016.108, *p* < 0.001, $\eta_{p}^{2}$ = 0.983). Post hoc comparisons showed a significant difference (all *p* < 0.01) between all levels of simulation apart from level 5 (*M* ± SD, 0.008 ± 0.029) and level 6 (0.000 ± 0.000; *p* = 0.762), where the majority of participants scored zero CS. VA and CS had a significant negative correlation (*r* = −0.914, *p* < 0.01).

### 3.2 Trial order and repetition

There was no significant main effect of trial order on outcome (*χ* ^2^(2) = 2.659, *p* > 0.05, *W* = 0.053). There was no significant main effect of trial repetition on outcome (*χ* ^2^(2) = 3.402, *p* > 0.05, *W* = 0.068).

### 3.3 Penalty performance

#### 3.3.1 Outcome

Missed and saved penalties were grouped for analysis of goals scored compared to not scored across vision conditions. There was a significant main effect of vision conditions on outcome (*χ* ^2^(7) = 14.607, *p* = 0.041, *W* = 0.083), with significantly fewer goals scored at level 6 (21 scored) compared to habitual (38 scored) (*Z* = −2.785, *p* = 0.005). Participants were 45% less likely to score a penalty at level 6 and level 7 compared to habitual vision. Despite the reduction in the number of goals scored at level 7 (21 scored) compared to habitual vision condition (*Z* = −2.450, *p* = 0.014), this did not reach the adjusted Bonferroni corrected significance level (*p* = 0.007).

**FIGURE 1:**
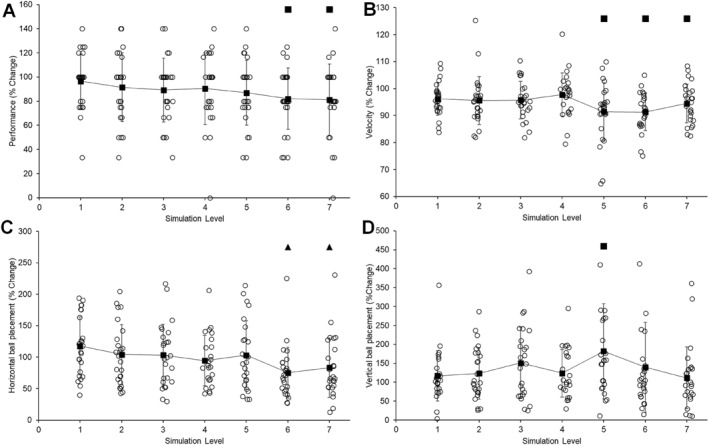
Comparing percentage change at each simulation level for all dependent variables (performance (A), velocity (B), horizontal ball placement (C) and vertical ball placement (D)) compared to habitual (control condition); ■ denotes significantly poorer performance compared to habitual (*p* < 0.05) and ▲ denotes significantly poorer performance compared to level 1 (*p* < 0.05).

There was a significant effect of simulation level on performance (*F*(7, 168) = 2.973, *p* = 0.006, $\eta_{p}^{2}$ = 0.110). While performance decreased descriptively across each level of simulation, post hoc comparisons only showed a significant decrease from habitual performance of 17.93% with level 6 (*p* = 0.026) and 18.87% with level 7 (*p* = 0.015) (Figure 1A).

#### 3.3.2 Initial ball velocity

There was a significant main effect of vision conditions on initial ball velocity (*F*(7, 175) = 6.130, *p* < 0.001, $\eta_{p}^{2}$ = 0.197). There was a 12% reduction in ball velocity at level 5 (17 ± 3 m s^−1^) and level 6 (17 ± 3 m s^−1^) when compared to habitual (19 ± 3 m s^−1^) vision condition (*p* = 0.035, *d* = 0.58 and *p* < 0.001, *d* = 0.56) (Figure 1B). There was a 6% reduction in ball velocity at level 7 when compared to habitual vision condition (*p* = 0.033, *d* = 0.37). There was no significant effect of repetition (*F*(2, 50) = 0.097, *p* > 0.05, $\eta_{p}^{2}$ = 0.004), or vision‐by‐repetition interaction effect (*F*(14, 350) = 1.709, *p* > 0.05, $\eta_{p}^{2}$ = 0.064).

#### 3.3.3 Horizontal ball placement

There was a significant effect of simulation level on how far from the centre of the goal participants placed PKs (*F*(7, 168) = 3.003, *p* = 0.005, $\eta_{p}^{2}$ = 0.111). Post hoc comparisons did not display a difference in ball location compared to the habitual condition for any level of simulation (all *p*'s > 0.05). There was a significant decrease of 42.38% in the distance from the centre of the goal between level 1 and 6 (*p* = 0.004) and 34.46% from level 1 to level 7 (*p* = 0.048) (Figure 1C).

#### 3.3.4 Vertical ball placement

There was a significant effect of simulation level on how high participants placed the ball in the goal (*F*(7, 168) = 3.167, *p* = 0.004, $\eta_{p}^{2}$ = 0.117). Post hoc comparisons did not display a significant difference in ball location compared to the habitual condition for any level of simulation (all *p*'s > 0.05) apart from a 74.769% increase in vertical ball placement for level 5 simulation (*p* = 0.005) (Figure 1D).

### 3.4 ROCs curves

The Youden's *J* for VA indicated that the greatest balance of sensitivity (0.75) and specificity (0.41) were at 1.54 logMAR (Youden's *J* = 0.19) (as shown in Figure S2A). Youden's *J* for CS indicated the greatest balance of sensitivity (0.47) and specificity (0.68) at 0.4 logCS (Youden's *J* = 0.15) (as shown in Figure S2B).

### 3.5 Decision tree analysis

Decision tree analysis revealed the first binary split at a VA of 1.61 logMAR, indicating that performance becomes ‘below expected’ past this level of VI. The pruned decision tree excluded CS. This indicates a similar cut‐off point as the ROC analysis that suggested a VA of 1.54 logMAR, whilst suggesting that CS is a poor predictor of performance (as shown in Figure S3).

## 4 EXPERIMENT 1 DISCUSSION

Experiment 1 aimed to identify the level at which simulated binocular VI inhibited placement, velocity and performance in a PK. Findings supported previous literature that performance was resilient to relatively high levels of simulated blur (Bulson et al., 2015; Mann et al., 2007, 2010; Vera et al., 2020), with strategy remaining the same as habitual until 1.61 logMAR. The first meaningful decrements in performance occurred when vision was degraded to 1.96 logMAR, which coincided with strategic differences (lower ball velocity and more central ball placement). ROC analysis revealed the best VA cut‐off within the data would be 1.54 logMAR (sensitivity = 0.75, specificity = 0.41; Youden's *J* = 0.16), with a CS of 0.40 logCS (sensitivity = 0.47, specificity = 0.68; Youden's *J* = 0.15). Decision tree analysis revealed an optimal cut‐off of 1.61 logMAR, a similar level to the ROC curve. However, VA and CS produced a low Youden's *J* value, indicating that binocular vision does not have a strong relationship with PK performance at these levels of impairment.

The reduced VF condition affected performance due to a lower ball velocity and caused participants to shoot closer to the centre of the goal. Previous research studies have suggested that peripheral vision plays a pivotal role during PKs. Where footballers typically fixate on the ball when taking a penalty, peripheral vision is associated with the collation of anticipatory cues, as players have reported fixating on the ball rather than the intended location, to limit a goalkeeper's ability to predict ball direction (Millard et al., 2022; Tedesqui & Orlick, 2015). However, under a restricted VF, participants were likely unable to employ this approach. Future work on VF in VI football performance is needed to build on the single condition investigated here.

The findings indicated that excessive binocular blur negatively impacted one's performance (Bulson et al., 2015; Mann et al., 2007). At 1.96 logMAR, the ball was struck at a lower velocity and placed closer to the target's midpoint. Although optimal visual clarity in participants were not critical in maintaining habitual performance, individuals were unable to compensate effectively at 1.96 logMAR. This is a similar VI severity as basketball and golf (Bulson et al., 2008, 2015), indicating a consistent point where individuals were unable to accrue visual information. This appears to represent a point where individuals are unable to adapt to maintain self‐paced sporting performance. Performance and strategy in this task were resilient past the current football MIC (1.00 logMAR). However, other elements for football performance, such as ball control, passing and dribbling, are affected at less severe levels of impairment (Runswick et al., 2023). This suggested that penalty performance is less relevant for MIC research but there is a possibility that a 1.96 logMAR may indicate a possible split between the partially sighted (B2/B3) and blind (B1) forms of the game as a highly resilient skill is negatively impacted by loss of vision at this point.

## 5 EXPERIMENT 2 INTRODUCTION

In experiment 1, we showed that high binocular blur levels negatively affected PK performance. However, this does not account for impairments that cause individuals to view their environment through two dissimilar images: monocular impairments (dominant eye occluded or blurred). Research examining other sporting tasks has indicated that monocular impairments may profoundly affect sporting performance (Bulson et al., 2015; Bulson & Ludlam, 2009; Vera et al., 2020). Currently, VI football classification is conducted through monocular assessment, and competitors are classified based on their best‐performing eye. Therefore, understanding the influence of binocular and monocular impairments on football performance will provide an evidence‐based rationale to justify classification procedures by providing insights into whether competitors should be classified monocularly or binocularly, as suggested by VI football experts (Runswick et al., 2021). The study aims to document the effects of monocular impairment on PK performance whilst comparing how different VIs (binocular vs. monocular) influence performance.

## 6 EXPERIMENT 2 METHODS

### 6.1 Participants

Twenty eight participants aged 20 ± 3 years were recruited. 75% of participants were right‐eye dominant and 25% left‐eye dominant. Participants had competed for the university futsal or football teams (*n* = 18), semi‐pro (*n* = 2) or were competing in organised amateur leagues (*n* = 8). Participants were deemed ineligible if they wore glasses or were injured. The university ethics panel ethically approved the study. Written informed consent was obtained and the research was conducted in accordance with the principles of the Declaration of Helsinki. Twenty‐four males and 4 females were recruited as classification evidence must reflect the impairment–performance relationship of the men's and women's games. We did not expect sex to alter the impairment–performance relationship (Runswick et al., 2023), as previously evidenced in VI Judo (Krabben, Mashkovskiy, et al., 2021). The women's game has recently been introduced at the 2023 IBSA world games, where 22% of footballers were female, indicating that our sample is an accurate representation of the study's target population.

### 6.2 Protocol

A block randomised study design was implemented in experiment 2, the same to that of experiment 1. Participants and goalkeepers were given identical instructions as those detailed in experiment 1, the protocol was completed identically, and the protocol was adhered to, resulting in no penalties being retaken. In total 24 PKs were taken (x3 per condition). Two goalkeepers of similar experience (active players competing in organised amateur leagues) were used during data collection. The same goalkeeper was used for every PK within a participant's dataset (goalkeeper only changed between participants). Goalkeepers only changed between participants when the primary goalkeeper was not available and this was due to practicalities around data collection days. These were different goalkeepers to those used in experiment 1.

### 6.3 Apparatus

The location of data collection and equipment used was the same to ensure that comparisons could be made across studies.

### 6.4 Simulation of VI

An adapted version of the porta eye (point‐a‐finger test) dominance test was implemented. Participants were instructed to extend both arms, with their index finger aiming directly forward toward a distant object (approx. 6 m) (Li et al., 2010). The eye that the index finger aligned with was deemed the dominant eye. Vision tests were conducted in the same laboratory and lighting conditions as the experimental procedure took place. During the vision tests (VA/CS) the non‐dominant eye was occluded (through wearing an eye patch) and the dominant eye was blurred from level 1–6, at level 7 the dominant eye was occluded causing the participants to view their environment through one eye (their non‐dominant eye). Whereas, during the PKs for levels 1–6 the eye patch was removed, resulting in a blurred eye (dominant eye) and a plano eye (non‐dominant eye).

The filters used were identical to experiment 1. Table 2 shows the seven levels of simulation, plus a control condition (habitual), how they were produced, and the mean levels of VA and CS they produced on established measures.

**TABLE 2:** Level of simulated monocular impairment (non‐dominant eye occluded during vision tests) used in this study.

Level	How simulated	Mean visual acuity logMAR (ETDRS)	Mean contrast sensitivity logCS (MARS)
Habitual	No simulation	−0.11 ± 0.07	1.78 ± 0.04
Level 1	Four layers of 258	0.79 ± 0.13	0.62 ± 0.13
Level 2	Five layers of 258	1.09 ± 0.17	0.36 ± 0.14
Level 3	Six layers of 258	1.33 ± 0.15	0.15 ± 0.11
Level 4	Seven layers of 258	1.58 ± 0.20	0.02 ± 0.04
Level 5	One layer of 256	1.73 ± 0.11	0.01 ± 0.03
Level 6	One layer of 253	1.99 ± 0.10	0.01 ± 0.03
Level 7	Monocular viewing (dominant eye occluded)	−0.03 ± 0.14	1.69 ± 0.10

### 6.5 Data analysis

The data analysed in experiment 2 was subject to identical data handling of experiment 1 to allow for direct comparisons between each study.

### 6.6 Statistical analysis

Statistical analysis of performance, learning/order effects, ball velocity and placement were identical in experiment 1. A student's independent samples *t*‐test was conducted to compare monocular and binocular PK performance. The VF condition (Exp 1, level 7) and habitual vision (Exp 2, habitual) were removed from the analysis, allowing comparison of no blur and direct comparisons of the same filters. The effect sizes were calculated using Cohen's *d*.

## 7 EXPERIMENT 2 RESULTS

### 7.1 Simulated impairments

There was a significant main effect of simulation level on VA (*F*(7, 189) = 130.794, *p* < 0.001, $\eta_{p}^{2}$ = 0.980). Post hoc analysis revealed that all vision conditions differed significantly (*p* < 0.001). CS comparisons indicated a significant main effect for CS simulations (*F*(7, 189) = 2297.620, *p < *0.001, $\eta_{p}^{2}$ = 0.988). Post hoc analysis indicated significant differences for all levels other than level 4 (0.02 ± 0.04), level 5 (0.01 ± 0.03) and level 6 (0.01 ± 0.03), where the majority of participants scored zero. VA and CS were significantly negatively correlated (*r* = −0.950, *p* < 0.001).

### 7.2 Trial order and repetition

There was no order effect on PK outcome (*χ* ^2^(2) = 2.835, *p* = 0.242, *W* = 0.051). There was no repetition effect on trial repetition for PK outcome (*χ* ^2^(2) = 5.856, *p* = 0.054, *W* = 0.105).

### 7.3 Penalty performance

#### 7.3.1 Outcome

Similar to experiment 1, penalties were grouped into scored and missed (saved/missed the target). Performance comparisons revealed there was no main effect of simulation level on penalty outcome (*F*(7, 189) = 0.850, *p* = 0.547, $\eta_{p}^{2}$ = 0.031). The most successful condition was level 7, where 53 penalties were scored (15% increase), compared to 46 for the habitual condition. The weakest performing condition was the level 6 condition, where 41 (11% decrease) penalties were scored.

**FIGURE 2:**
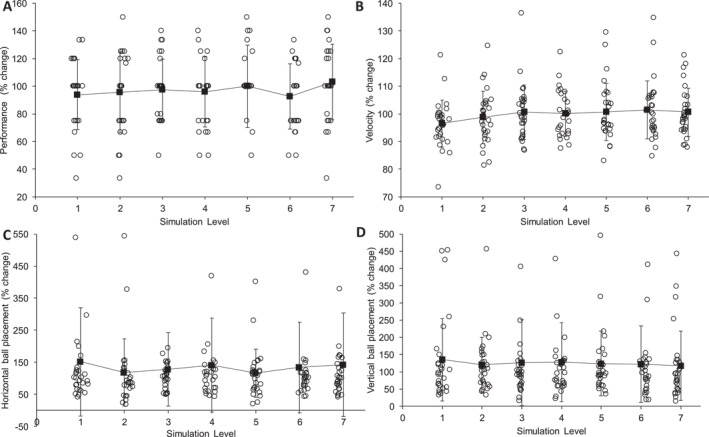
Comparing percentage change at each simulation level for all dependent variables (performance (A), velocity (B), horizontal ball placement (C) and vertical ball placement (D)) compared to habitual (control condition).

Relative performance comparisons revealed no main effect for each level of simulation (*F*(7, 189) = 0.771, *p* = 0.613, $\eta_{p}^{2}$ = 0.028) (Figure 2A). Descriptive analysis revealed a 3% increase in performance at level 7 compared to habitual performance, which was the strongest performing simulation. The poorest performance occurred at level 6, indicating a 7% decrease in performance compared to habitual.

#### 7.3.2 Initial ball velocity

Statistical comparisons revealed no main effect when comparing initial ball velocity in each level of simulation (*F*(5.000, 135.012) = 2.050, *p* = 0.076, $\eta_{p}^{2}$ = 0.071) (Figure 2B). The greatest difference compared to habitual was a 3.95% velocity reduction within level 1. There were no repetition effect (*F*(2, 54) = 0.050, *p* = 0.951, $\eta_{p}^{2}$ = 0.002), or vision‐by‐repetition interaction effect observed (*F*(14, 378) = 1.181*, p* = 0.287, $\eta_{p}^{2}$ = 0.042).

#### 7.3.3 Horizontal ball placement

There was no main effect when comparing horizontal ball placement across each level of simulation (*F*(1.966, 53.090) = 1.669, *p* = 0.199, $\eta_{p}^{2}$ = 0.058) (Figure 2C). Statistical comparison of repetition revealed no differences (*F*(2, 54) = 0.363*, p* = 0.697, $\eta_{p}^{2}$ = 0.013).

#### 7.3.4 Vertical ball placement

There was no main effect of simulation level on vertical ball placement (*F*(3.373, 91.084) = 0.805, *p* = 0.507, $\eta_{p}^{2}$ = 0.029) (Figure 2D). No repetition effect was identified for vertical ball placement (*F*(2, 54) = 2.743, *p* = 0.697, $\eta_{p}^{2}$ = 0.092).

### 7.4 Monocular versus binocular performance

Monocular performance (97.02 ± 3.03%) was significantly closer to habitual performance than artificial binocular impairment (90.98 ± 5.50%) *t*(369.000) = 2.274, *p* = 0.02, *d* = 0.237 (as shown in Figure S5). These findings indicate that under artificial binocular impairment conditions, participants could not maintain habitual performance.

## 8 EXPERIMENT 2 DISCUSSION

In experiment 2, we aimed to investigate the effects of monocular impairment on PK performance and compare this to binocular effects from experiment 1. Participants were able to adapt and acquire sufficient visual information and maintain performance in the task irrespective of the blur severity. This contradicts previous findings, where monocular impairments typically profoundly affect performance due to an inferior ability to perceive depth (stereopsis) (Bulson & Ludlam, 2009; Vera et al., 2020). Vera et al. (2020) reported reductions in performance during monocular viewing/impairments, which could be attributed to using a task that has a greater reliance on one's ability to perceive depth, amplifying the impairment effect on performance. Contradictory findings could be attributed to the manipulation of target area (size) (Bulson et al., 2008) and examination of smaller target areas (e.g. basketball hoop; Vera et al., 2020) where task precision and depth perception demands are elevated. These findings indicate that thresholds for where performance is influenced appear to be conflicting and task‐dependent. In comparison, when taking a PK, the ball is closer to the individual. This could be considered a ‘near target’, where the depth perception task demands are reduced, potentially accounting for the conflicting findings. Performance was maintained despite degrading or eliminating an individual's ability to perceive depth; this indicates that the task of a PK has little reliance on the ability to perceive depth, resulting in a high tolerance to artificial monocular impairment.

Results implied that individuals who suffer from monocular conditions (strabismus, anisometropia or aniseikonia) would not be disadvantaged when performing a PK, providing their other eye was of normal functionality. Alongside minimal depth perception demands an additional explanation for artificial monocular impairments not deteriorating PK performance could be attributed to the greater room for error (larger target area). This demonstrating task complexity directly influences the effects of visual blur and monocular impairments. This adds further evidence that the impact of VI is not just dependent on the sport but task‐dependent within the same sport.

## 9 GENERAL DISCUSSION

This two‐experiment study aimed to identify if futsal PK performance is affected by binocular and monocular blur and, if so, document the earliest point at which velocity, placement, and overall penalty performance deteriorates. The findings revealed that performance displayed a high resilience to both binocular and monocular visual blur and that VI does not have a strong relationship with PK performance until it is at severe levels of around 1.96 logMAR. Participants in experiment 1 were resilient to relatively high levels of binocular blur, with excessive impairments eventually altering performance and strategy. Monocular impairment in experiment 2 reported no significant differences in performance from the habitual level. These findings indicate that optimal VA and CS are not crucial to maintain habitual performance in this task. Moreover these findings add further evidence that thresholds at which the performance is impacted appear to be conflicting and task‐dependent (Bulson et al., 2015; Vera et al., 2020), with self‐paced motor skills indicating a higher resilience to VI, whereas interceptive and dynamic tasks display greater sensitivity to VI (Mann et al., 2007, 2010; Runswick et al., 2023).

A notable difference between binocular and monocular impairment was the ability to maintain velocity and placement. Participants implemented the same velocity and ball placement even when the dominant eye was excessively blurred. However, this was negatively affected by excessive binocular blur. This supports the notion that it may be beneficial to classify VI footballers through binocular assessments as this is likely to be a more accurate measure of vision used during a match, as suggested by the expert panel in Runswick et al. (2021). Our findings show that artificial binocular blur is less robust compared to artificial monocular impairments when taking PKs, a self‐paced motor skill performed under minimal time constraints, nor is the ball moving or performed under defensive pressure. However, these findings must be interpreted cautiously and carefully applied to other skills in football. Future work must investigate the impact of artificial monocular impairments in dynamic situations that recreates an open play scenario (potentially utilising the VIFs test (Runswick et al., 2023)). As previous work has shown a detrimental influence on multiple object tracking tasks under artificial monocular impairments within scenarios of dynamically moving objects (Zwierko et al., 2024). Moreover, artificial binocular impairment in football has shown a greater sensitivity when assessing dynamic technical skills (passing, dribbling and ball control (Runswick et al., 2023)) compared to the present study, which are skills that are likely to be implemented more frequently during competition.

CS was used to assess functional vision alongside VA in both experiments. CS was a poor predictor of penalty performance, as evidenced by the weak Youden's *J* values. Decision tree analysis supported these findings, as the model solely classified individuals through VA alone whilst excluding CS. These findings support the previous evidence that has investigated the effects of CS on other aspects of football performance (Runswick et al., 2023). As in previous research studies, this could be attributed to the nature of CS tests where scores of 0.00 logCS are recorded regularly when participants are still able to perform a task (Runswick et al., 2023). Within this study, VA and CS were highly correlated; therefore, VA could be considered a strong measure of multiple visual functions, similar to the findings within VI judo (Krabben, Ravensbergen, et al., 2021). Combined with Runswick et al. (2023), these findings indicate that CS is a poor predictor of football performance and that CS should be used cautiously in dynamic sports such as football. These findings support the previous work in Judo (Krabben, Ravensbergen, et al., 2021), where CS was also deemed a poor predictor of performance.

While findings from this study do extend the knowledge of the impairment–performance relationship in football, findings from this study should be applied cautiously. PKs are self‐paced tasks of minimal complexity and just one aspect of a sport considered a complex dynamic game of interacting variables (skills). However, this work expanded on previous VI football simulations that assessed technical and anticipatory performance through the implementation of the VIFS test and suggested that other elements of performance are affected much earlier than penalties (Runswick et al., 2023). Here, we also added the element of VF, but only through a single tunnel vision simulation. The effects of this severe field loss warrants further investigation with systematic studies of VF in football, particularly due to the potential importance of peripheral vision in such a dynamic sport (Vater et al., 2019, 2020) and experts opinions on the influence of reduced VF on performance (Runswick et al., 2021).

Future works should also explore these findings within individuals with VI as opposed to inducing artificial impairment. Testing a wide range of individuals will also allow optimal class structures to be designed by assessing those competing within the sport. Moreover, inducing a systematic reduction in VF would enable its impairment‐performance relationship to be explored, enabling the establishment of the true effect of degraded VF on football performance to be understood. Simulation studies would also enable VFs below and beyond the current paralympic inclusion criteria to be assessed, providing insights into the MIC and class structure. Moreover, the influence of monocular impairments on other key football skills (those measured within the VIFs test) or within realistic gameplay scenarios could be explored to understand the full effect of the monocular impairment–performance relationship.

In conclusion, PK performance was robust to severe VI but was eventually affected by loss of binocular but not monocular VA. This occurred at a much more severe level of impairment than other elements of football performance; however, it may be more relevant in the development of class structure than the MIC. This study adds further evidence that VA is a superior predictor of football performance compared to CS and suggests that it may be more appropriate for the classification procedure to incorporate binocular assessments.

## CONFLICT OF INTEREST STATEMENT

No conflict of interest to disclose.

## Supporting information

Supporting Information S1

## Data Availability

https://osf.io/evrwb/?view_only=9ba9b992644c477e95fb947daba59c28.
