# Supplementary material for: The effects of simulated monocular and binocular vision impairment on football penalty kick performance
Source: Eur J Sport Sci. 2024 Jun 8;24(7):918–29. doi: 10.1002/ejsc.12145 (PMC11235806; doi:10.1002/ejsc.12145)
Supplement: Supplementary file 1 — Supporting Information S1 [file EJSC-24-918-s001.docx]

**Supplementary Material:** The effects of simulated monocular and binocular vision impairment on football penalty kick performance

**Experiment 1**

**Simulated Impairments**

Figure 1. VA (logMAR) and CS (logCS) at each simulation level for binocular Impairment, each condition listed in the legend.

**Penalty Performance**

Table 1. The number of penalties scored in experiment 1 displaying absolute and relative values.

|  | **Scored** | **Missed** | **Saved** | **Total** |
| --- | --- | --- | --- | --- |
| Penalties (n) | 235 | 314 | 51 | 600 |
| Percentage (%) | 39% | 52% | 9% | 100% |

**Receiver Operator Characteristics (ROC) Curves
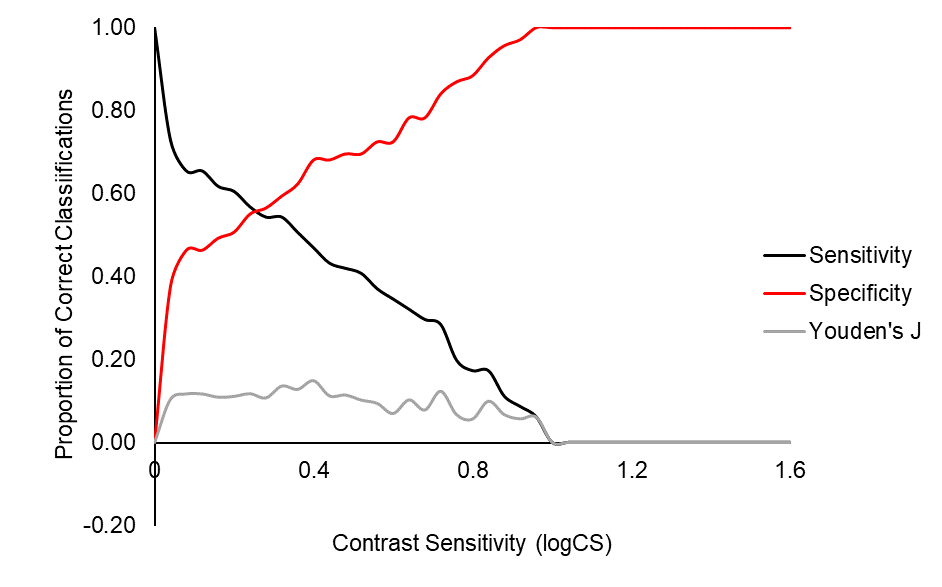

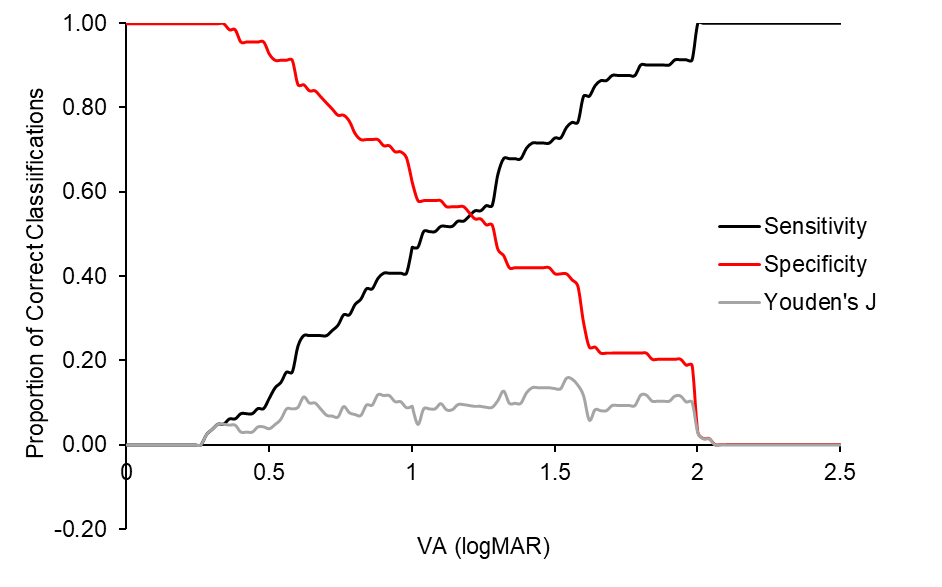
**

**B**

**A**

Figure 2. (A) The sensitivity and specificity for the MIC for VA and PK performance (B) The sensitivity and specificity for the MIC for CS and PK performance

**Decision Tree Analysis**

**
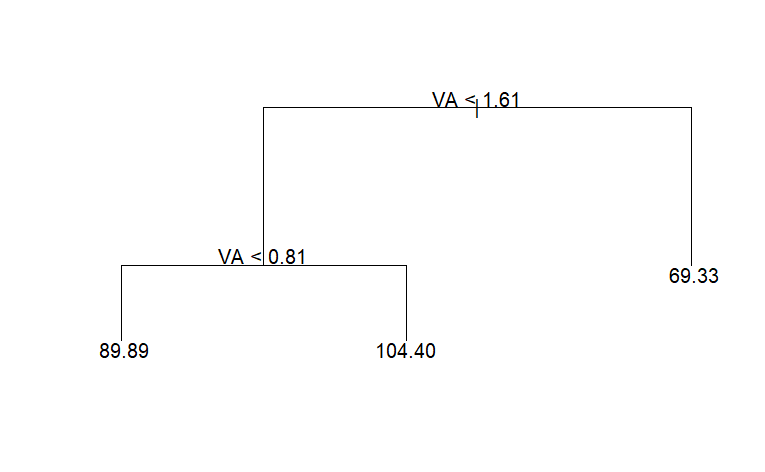

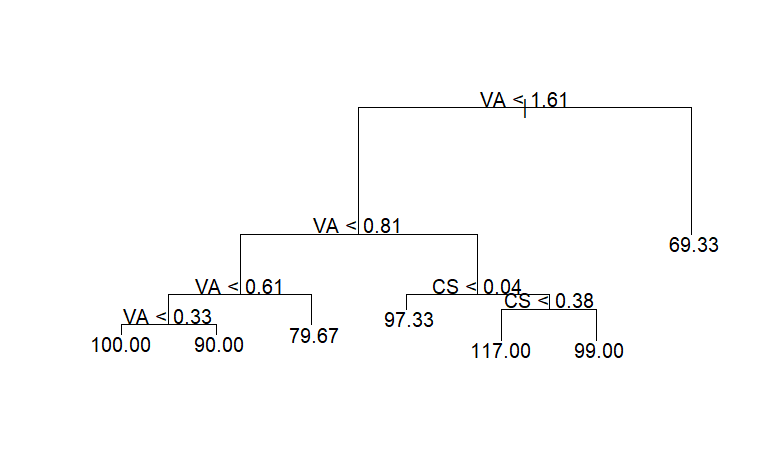
**Figure 3. Decision tree model for MIC when classifying individuals on PK performance. (A) Full decision tree model (B) the pruned decision tree model.

**A**

**B**

**Experiment 2**

**Simulated impairments**

Figure 4. VA (logMAR) and CS (logCS) at each simulation level for monocular Impairment, each condition listed in the legend.

**Penalty performance**

Table 2. The number of penalties scored in experiment 1 displaying absolute and relative values.

|  | **Scored** | **Missed** | **Saved** | **Total** |
| --- | --- | --- | --- | --- |
| Penalties (n) | 366 | 221 | 85 | 672 |
| Percentage (%) | 54% | 33% | 13% | 100% |

Figure 5. Performance measure as a percentage of habitual performance for each simulation level across monocular (Exp 2) vs. binocular (Exp 1).
